# Heterogeneous and Flexible Transmission of *mcr-1* in Hospital-Associated Escherichia coli

**DOI:** 10.1128/mBio.00943-18

**Published:** 2018-07-03

**Authors:** Yingbo Shen, Zuowei Wu, Yang Wang, Rong Zhang, Hong-Wei Zhou, Shaolin Wang, Lei Lei, Mei Li, Jiachang Cai, Jonathan Tyrrell, Guo-Bao Tian, Congming Wu, Qijing Zhang, Jianzhong Shen, Timothy R. Walsh, Zhangqi Shen

**Affiliations:** a Beijing Advanced Innovation Center for Food Nutrition and Human Health, College of Veterinary Medicine, China Agricultural University, Beijing, China; b Beijing Key Laboratory of Detection Technology for Animal-Derived Food Safety and Beijing Laboratory for Food Quality and Safety, Beijing, China; c College of Veterinary Medicine, Iowa State University, Ames, Iowa, USA; d Second Affiliated Hospital of Zhejiang University, Zhejiang University, Hangzhou, China; e Department of Medical Microbiology and Infectious Disease, Institute of Infection & Immunity, Heath Park Hospital, Cardiff, United Kingdom; f Zhongshan School of Medicine, Sun Yat-Sen University, Guangzhou, China; University of Wisconsin—Madison

**Keywords:** E. coli, genetic diversity, *mcr-1*, population genomics, transmission

## Abstract

The recent emergence of a transferable colistin resistance mechanism, MCR-1, has gained global attention because of its threat to clinical treatment of infections caused by multidrug-resistant Gram-negative bacteria. However, the possible transmission route of *mcr-1* among *Enterobacteriaceae* species in clinical settings is largely unknown. Here, we present a comprehensive genomic analysis of Escherichia coli isolates collected in a hospital in Hangzhou, China. We found that *mcr-1*-carrying isolates from clinical infections and feces of inpatients and healthy volunteers were genetically diverse and were not closely related phylogenetically, suggesting that clonal expansion is not involved in the spread of *mcr-1*. The *mcr-1* gene was found on either chromosomes or plasmids, but in most of the E. coli isolates, *mcr-1* was carried on plasmids. The genetic context of the plasmids showed considerable diversity as evidenced by the different functional insertion sequence (IS) elements, toxin-antitoxin (TA) systems, heavy metal resistance determinants, and Rep proteins of broad-host-range plasmids. Additionally, the genomic analysis revealed nosocomial transmission of *mcr-1* and the coexistence of *mcr-1* with other genes encoding β-lactamases and fluoroquinolone resistance in the E. coli isolates. These findings indicate that *mcr-1* is heterogeneously disseminated in both commensal and pathogenic strains of E. coli, suggest the high flexibility of this gene in its association with diverse genetic backgrounds of the hosts, and provide new insights into the genome epidemiology of *mcr-1* among hospital-associated E. coli strains.

## IMPORTANCE

Colistin represents one of the very few available drugs for treating infections caused by extensively multidrug-resistant Gram-negative bacteria. The recently emergent *mcr-1* colistin resistance gene threatens the clinical utility of colistin and has gained global attention. How *mcr-1* spreads in hospital settings remains unknown and was investigated by whole-genome sequencing of *mcr-1*-carrying Escherichia coli in this study. The findings revealed extraordinary flexibility of *mcr-1* in its spread among genetically diverse E. coli hosts and plasmids, nosocomial transmission of *mcr-1*-carrying E. coli, and the continuous emergence of novel Inc types of plasmids carrying *mcr-1* and new *mcr-1* variants. Additionally, *mcr-1* was found to be frequently associated with other genes encoding β-lactams and fluoroquinolone resistance. These findings provide important information on the transmission and epidemiology of *mcr-1* and are of significant public health importance as the information is expected to facilitate the control of this significant antibiotic resistance threat.

## INTRODUCTION

The relentless increase in the populations of multidrug-resistant (MDR) and extensively drug-resistant (XDR) Gram-negative bacterial strains is worrying, not least because we apparently have no new clinical options. Moreover, the antibiotic pipeline is bereft of novel entities to potentially cover MDR and XDR Gram-negative infections (1–4). Recent global attention has focused on the plight of our entry into the “postantibiotic era” in the face of the rapid dissemination of carbapenem-resistant mechanisms (NDM-1, KPC, and OXA-48/181/232) and the realization of the very limited number of antibiotics, e.g., colistin, that we have left to treat serious infections (5–7). Until recently, colistin resistance was observed to be mediated by chromosomal mutations only and commonly occurred in Klebsiella pneumoniae and Pseudomonas aeruginosa but rarely in Escherichia coli (8). However, the first transferable colistin resistance mechanism, termed *mcr-1*, was recently reported in *Enterobacteriaceae* from both food-producing animals and human origins, in particular, E. coli (9). Perhaps of greater concern is the coexistence of *mcr-1* and carbapenem resistance genes, such as *bla*_NDM-5/9_ and *bla*_KPC-2_, recently identified in E. coli from human infections and poultry production (10–13), as the common occurrence of the *mcr-1* gene in carbapenem-resistant *Enterobacteriaceae* (CRE) would seriously compromise current treatment options not just in China but globally.

The unprecedented global increase in the populations of CRE, and now of *mcr-1*-positive *Enterobacteriaceae* (MCRPE), has placed further pressure on drug discovery programs to produce novel antimicrobials. It is still unclear what drives CRE and MCRPE, and although the increase in antibiotic consumption (e.g., the use of carbapenems) has been attributed to this increase, the remarkable plasticity and fluidity of DNA structures in Gram-negative bacteria have made a significant contribution. In *Enterobacteriaceae*, this horizontal gene transfer is fueled by a potent cocktail of plasmids, transposons, insertion sequence (IS) elements, and insertion sequence common region (ISCR) elements (14, 15). For example, the *bla*_NDM-1_ gene can be found in considerably greater numbers of bacteria than its KPC counterpart—in part, as a result of the diversity of plasmids it is associated with (16). The immediate genetic context surrounding the *bla*_NDM-1_ gene is also remarkably heterogeneous and has contributed to its translocation between chromosome and plasmid, and vice versa, and between plasmids (16).

Due to its global significance, many studies have been reported on investigating the prevalence of *mcr-1* and have characterized its genetic environments in *Enterobacteriaceae*. To date, more than 70 completed sequences of plasmids carrying *mcr-1* have been deposited into the GenBank database and their data show that they are relatively narrow in range and contain few other antibiotic resistance genes (11, 17–27). Thus far, many whole-genome sequences have been retrospectively searched for *mcr-1*; however, the majority of the reports focus only on the gene or plasmid and few have analyzed its associated bacterial hosts (18, 28). Moreover, data on the possible transmission routes of *mcr-1* among MCRPE are largely lacking.

In order to aid understanding of the prevalence and outcomes of the presence of MCRPE in patients as well as in healthy adults, two studies were recently published on *mcr-1*-positive isolates and their impacts on nosocomial infections (29, 30). Statistical data suggest that *mcr-1*-positive E. coli (MCRPEC) infections were found to be associated with male sex, immunosuppression, and antibiotic usage (30). Multiple studies have also shown that E. coli is the most frequently observed *Enterobacteriaceae* species carrying the *mcr-1* gene and that the gene can be transferred to other *Enterobacteriaceae* species from MCRPEC at high frequencies (9, 29, 30). However, despite those studies, there is a marked paucity of genetic data on MCRPE in hospitals that can help researchers to understand their circulation and thus their potential impact on infection control policies. Here, we present an extensive whole-genome analysis of 80 E. coli strains isolated from both clinical samples and fecal samples of patients and healthy human volunteers in one hospital in Hangzhou, Zhejiang Province, in China.

## RESULTS

### Overview of *mcr-1*-positive E. coli*.*

During our previous epidemiological and clinical study, we obtained a considerable number of MCRPEC isolates from patients and healthy adults in Hangzhou, Zhejiang, China (30). Here, we performed whole-genome analysis of 80 MCRPEC strains to address the possible dissemination of the *mcr-1* gene (see Table S1 in the supplemental material). Briefly, 36 MCRPEC isolates were derived from nosocomial infections (urinary tract, surgical wounds, respiratory tract, etc.) of inpatients (*n* = 2,577), while 27 were from fecal samples of inpatients (*n* = 1,028) and 17 were from fecal samples of healthy volunteers (*n* = 2,909) collected in 2015. Detailed clinical information on these 80 *mcr-1*-carrying E. coli isolates is presented in Table S2.

10.1128/mBio.00943-18.1 TABLE S1  The E. coli isolates in this study and their information from sequencing analysis. Download TABLE S1, XLSX file, 0.04 MB. Copyright © 2018 Shen et al. 2018 Shen et al. This content is distributed under the terms of the Creative Commons Attribution 4.0 International license.

10.1128/mBio.00943-18.2 TABLE S2  Clinical information on 80 E. coli isolates with the *mcr-1* gene and the MICs of multiple antimicrobial agents. Download TABLE S2, XLSX file, 0.05 MB. Copyright © 2018 Shen et al. 2018 Shen et al. This content is distributed under the terms of the Creative Commons Attribution 4.0 International license.

### Genome sequencing of the 80 *mcr-1*-positive E. coli isolates.

At least 100× coverage of raw reads from Illumina sequencing was obtained for each isolate. The draft genomes were assembled *de novo* using CLC Genomics Workbench (version 8.5). The number of contigs ranged from 51 to 232, while the N50 of contigs ranged from 43 kb to 521 kb (Table S1) for the isolates assembled by CLC Genomics Workbench (version 8.5). Since the majority of the *mcr-1* genes were located on the plasmids, we further used the plasmidSPAdes program to optimize the assembly of plasmids. The *mcr-1*-containing contigs for each isolate were extracted from the two assemblies, and the longer contig was used to determine the genetic context of *mcr-1*. The lengths of *mcr-1-*carrying contigs generated by Illumina sequencing ranged from 2,388 to 141,207 bp (Table S1). Due to the shortness of the reads generated by Illumina sequencing and the high number of insertion elements, the assembled *mcr-1*-carrying contigs for nine isolates were short (2.3 to 3.8 kb). Thus, these isolates were resequenced by single-molecule real-time (SMRT) sequencing to generate complete chromosomes and plasmids (Table S1 and S3). A further six isolates were also subjected to SMRT sequencing.

10.1128/mBio.00943-18.3 TABLE S3  The assembly stats for the PacBio genomes. Download TABLE S3, XLSX file, 0.03 MB. Copyright © 2018 Shen et al. 2018 Shen et al. This content is distributed under the terms of the Creative Commons Attribution 4.0 International license.

### Genomic epidemiology of the *mcr-1*-carrying E. coli isolates.

**FIG 1:**
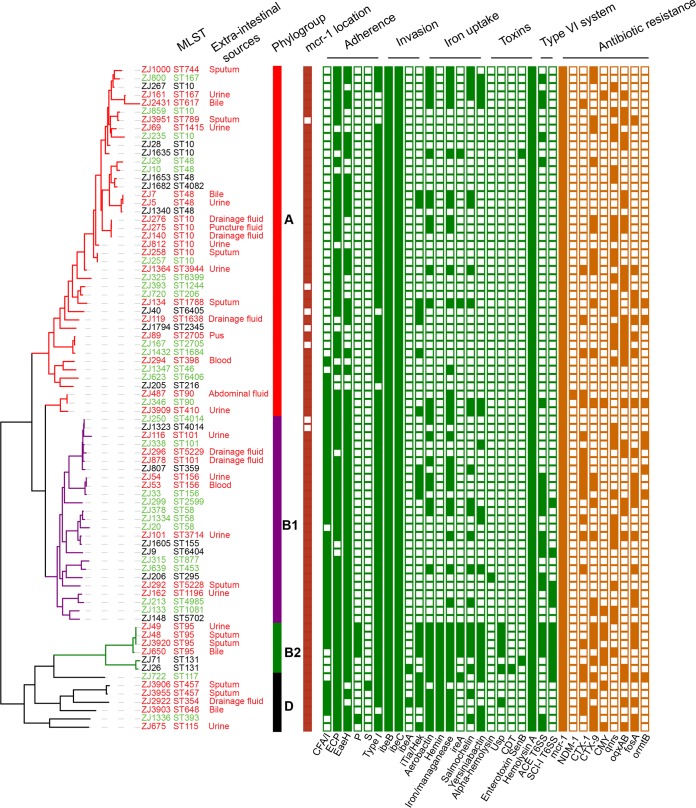
Genomic analysis of *mcr-1*-carrying E. coli isolates in a single hospital system in Zhejiang Province, China. A maximum-likelihood phylogenetic tree was constructed using the core genome SNPs and midpoint rooted. Sources of the isolates are indicated by different colors for strain identification (ID) plus MLST (red, infectious sample of inpatient; green, feces of inpatient; black, feces of healthy volunteer). E. coli phylogroups are denoted by colored strips, and the branches of the tree are colored in correspondence to the coloring of the strips. The location of *mcr-1* on a plasmid or chromosome (dark red) and the presence or absence of virulence genes (green) and antibiotic resistance genes (orange) are denoted by filled and empty squares, respectively. Only categories of the virulence genes and 9 clinically important antibiotic resistance genes are shown. Details of the genes in each category are given in Table S4 and S5.

The genomic and epidemiological relationships among all MCRPEC isolates were investigated (Fig. 1). Classification of strains to phylogenetic subgroups (31, 32) and of sequence types using multilocus sequence typing (MLST) (33) was performed through *in silico* analysis (Fig. 1). The core genome phylogenetic tree indicates 3 main clusters, but MLST analysis revealed that the 80 MCRPEC isolates were significantly diverse: 76 isolates were assigned to 46 known MLST types, and 4 other isolates (ST6399, ST6404, ST6405, and ST6406) possessed novel STs (Fig. 1). The most prevalent ST of isolates harboring *mcr-1* gene was ST10, accounting for 12.5% of all isolates. This ST is also commonly observed in extended-spectrum-β-lactamase (ESBL)-carrying E. coli isolates of both human and animal origins (34, 35). The classification of phylogenetic subgroups demonstrated that the 80 isolates were distributed throughout the four phylogroups (A, B1, B2, and D) (32), but the majority fell within the A and B1 groups, with 51.3% (*n* = 41) in the A group and 33.8% (*n* = 27) in the B1 group. Only 6.3% (*n* = 5), and 8.8% (*n* = 7) of the strains belonged to group B2 and group D, respectively (Fig. 1). Analyzing the etiology of group A and B1 isolates, we found that intestinal and extraintestinal isolates were extensively disseminated throughout each phylogenetic group, without significant enrichment in either of the phylogroups (*P* > 0.05 [Fisher’s exact test]). These results indicated that no clear phylogenomic division exists between intestinal strains and extraintestinal strains as observed in the disparate sequence types (such as ST10, ST48, ST156, etc.) (Fig. 1). Similar distributions were observed in groups B2 and D, although each group contained only a limited number of isolates.

### VFs and antibiotic resistance profiles of *mcr-1*-carrying isolates.

The virulence factors (VFs) in E. coli play an important role in conferring selective advantages and defining pathogenicity profiles. We therefore cataloged the known VFs, including genes associated with adherence, autotransporters, invasion, iron uptake, toxins, and secretion systems, as well as the antibiotic resistance phenotypes. The 12 isolates from phylogroups B2 and D possessed the greatest number of VFs, while 68 isolates of phylogroup A and B strains displayed low prevalence of VFs (Fig. 1; see also Table S4). However, no significant difference was observed in the frequency of VFs between the intestinal and extraintestinal isolates. Furthermore, no VFs related to intestinal pathogenic E. coli could be found, which is consistent with the clinical observation of the patients. Several genes encoding fimbriae were commonly present among the isolates, including those encoding E. coli common pilus (ECP), EaeH, and type 1 fimbriae; however, colonization factor antigen I (CFA/I) fimbriae were less frequently associated with phylogroup A. Types P and S fimbriae, related to extraintestinal infection, were found in phylogroups B2 and D (Fig. 1). Antibiotic resistance profiling showed that all strains were resistant to multiple categories of drugs, including colistin. Two isolates, ZJ134 (from sputum of an inpatient) and ZJ33 (from feces of an inpatient), were resistant to 11 classes of antimicrobials (Table S2); however, the resistance profiles of the isolates were not specific to any phylogenetic group. A total of 58.8% (47/80) of the isolates carried both *mcr-1* and ESBL genes (Table S5). Of particular note, one isolate, ZJ487 (ST90), from intra-abdominal fluid contained not only *mcr-1* but also *bla*_CTX-M-1_, *bla*_NDM-1_, *bla*_CTX-M-55_, *bla*_OXA-1_, and *bla*_SHV-12_. E. coli ST131 is the most prevalent sequence type associated with extraintestinal infections (36–38), and we identified two *mcr-1*-positive ST131 isolates of the serotype of H4:O25. One ST131 isolate, ZJ26, from the fecal sample of a healthy volunteer, carried VF genes *iroN*, *iha*, gad, *iss*, *cma*, *tsh*, and *fimH22* and antibiotic resistance genes *bla*_CTX-M-55_ and *mcr-1* and the *oqxAB* plasmid-mediated quinolone resistance gene. The other ST131 isolate, ZJ71, from the fecal sample of a healthy volunteer, possessed fluoroquinolone resistance alleles *gyrA1AB* and *parC1aAB*, *bla*_CTX-M-14_, *bla*_TEM-1B_, *tet*(A), and VF genes *sat*, *iss*, *iha*, and *senB*; the *fimH30*-*gyrA*1AB-*parC*1aAB allelic profile of that isolate was considered to show that it was the more pandemic of the two ST131 isolates (39).

10.1128/mBio.00943-18.4 TABLE S4  Characterization of virulence genes in 80 *mcr-1*-carrying E. coli isolates. Download TABLE S4, XLSX file, 0.1 MB. Copyright © 2018 Shen et al. 2018 Shen et al. This content is distributed under the terms of the Creative Commons Attribution 4.0 International license.

10.1128/mBio.00943-18.5 TABLE S5  Characterization of resistance genes in 80 *mcr-1*-carrying E. coli isolates. Download TABLE S5, XLSX file, 0.1 MB. Copyright © 2018 Shen et al. 2018 Shen et al. This content is distributed under the terms of the Creative Commons Attribution 4.0 International license.

### *mcr-1* is mainly plasmid mediated.

**FIG 2:**
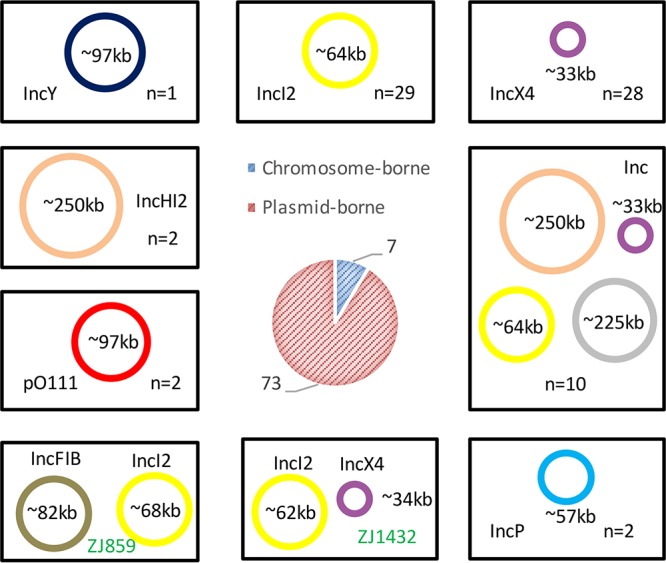
Schematic depiction of the prevalence of *mcr-1* in various types of plasmids or on the chromosomes. The types of plasmids potentially carrying the *mcr-1* gene were derived from the *mcr-1*-carrying contigs of each isolate. A colored circle represents a reference *mcr-1*-carrying plasmid of each type or the complete plasmid determined by SMRT sequencing in this study, and the size of the circle is proportional to that of the reference plasmid. Ten *mcr-1*-carrying plasmids whose replicons were not in the same contigs as *mcr-1* are grouped in one box (Inc). *mcr-1* is chromosome-borne in 7 isolates and plasmid-borne in 73 isolates (central pie chart). IncI2 and IncX4 are the major types of *mcr-1*-carrying plasmids. Isolates ZJ859 and ZJ1432 contained two *mcr-1*-carrying plasmids each. Thus, the total number of *mcr-1*-carrying plasmids is 75 in this study.

To classify the locations of *mcr-1* in the genomes, *mcr-1*-containing contigs were extracted from the complete genomes of 15 isolates subjected to SMRT sequencing and the draft genomes of 65 isolates sequenced by Illumina. The results of analysis of the complete genomes of 15 isolates suggested that *mcr-1* is located on the chromosome in 6 isolates and on plasmids in 9 isolates (Fig. 2; see also Table S1 and S3). Interestingly, isolates ZJ1432 and ZJ859 contain two copies of *mcr-1*, which were located on distinct plasmids (Fig. 2). *mcr-1* was carried on an ~34-kb IncX4 plasmid and an ~62-kb IncI2 plasmid in ZJ1432, while *mcr-1* was carried on an ~68-kb IncI2 plasmid and an ~82-kb IncFIB plasmid in ZJ859. To determine the locations of *mcr-1* in the rest of the 65 isolates sequenced by Illumina, *mcr-1*-containing contigs from the 65 isolates were searched against the PlasmidFinder database (40) to define their plasmid types, and the whole draft genomes of each of the isolates were subjected to a BLAST search against the complete *mcr-1*-carrying plasmids deposited in GenBank to find the closest reference. PlasmidFinder detected that 54 of the 65 *mcr-1*-positive contigs also carried plasmid replicons, suggesting that their *mcr-1* genes are plasmid-borne genes. The other 11 isolates did not possess any known plasmid replicons within the *mcr-1*-positive contigs. When the complete set of contigs for each isolate was mapped to reference plasmids, 10 of 11 isolates mapped more than 95% to their closest reference plasmids (Fig. 2; see also Table S1). Replicons of the 10 isolates were reexamined using the complete set of contigs of each genome. The results showed that all 10 isolates contain the same replicons as the reference plasmids (see Fig. S1 to S4 at https://doi.org/10.6084/m9.figshare.6281579.v1), but the replicons were separated from *mcr-1*-containing contigs due to the incomplete assembly. The only exception was the *mcr*-*1* contig (~19.7 kb) from isolate ZJ3951, which did not contain any plasmid replicons and did not map sufficiently with any known plasmids. However, that contig is highly similar to homologous regions of the E. coli chromosome available in the GenBank database (e.g., CP018962.1 and CP018103.1), indicating that *mcr-1* in ZJ3951 is located on the chromosome (see Fig. S5 at https://doi.org/10.6084/m9.figshare.6281579.v1). As expected, the locations of *mcr-1* based on the whole-genome sequence analysis were highly consistent with the S1 pulsed-field gel electrophoresis (PFGE) results (see Fig. S6 at https://doi.org/10.6084/m9.figshare.6281579.v1), represented as chromosome-borne or plasmid-borne. In total, *mcr-1* was found to be chromosome-borne in 7 isolates and plasmid-borne in 73 isolates. As indicated in the NCBI database, IncI2 (*n* = 29, 38.7%) and IncX4 (*n* = 28, 37.3%) were the two main plasmid types carrying *mcr-1* gene (Fig. 2). A 97-kb p0111 plasmid was first found to carry the *mcr-1* gene by SMRT sequencing in two isolates, ZJ275 and ZJ276.

### Genetic context of *mcr-1* in the 80 E. coli isolates.

**FIG 3:**
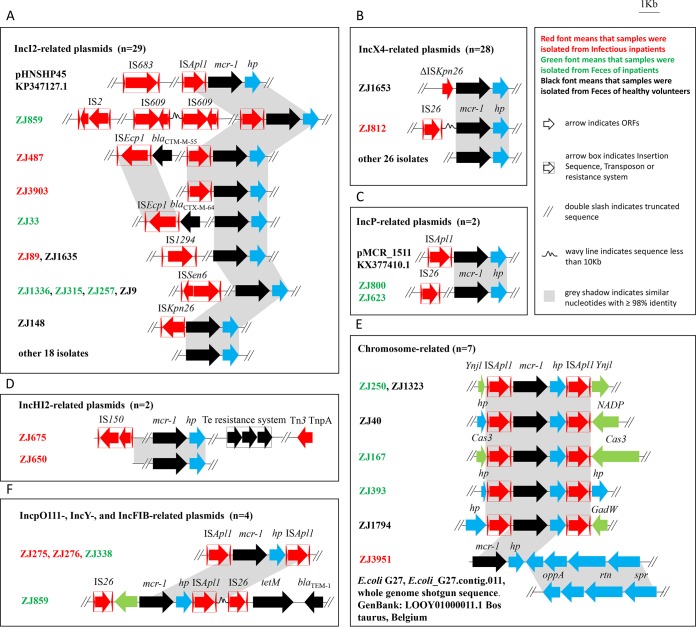

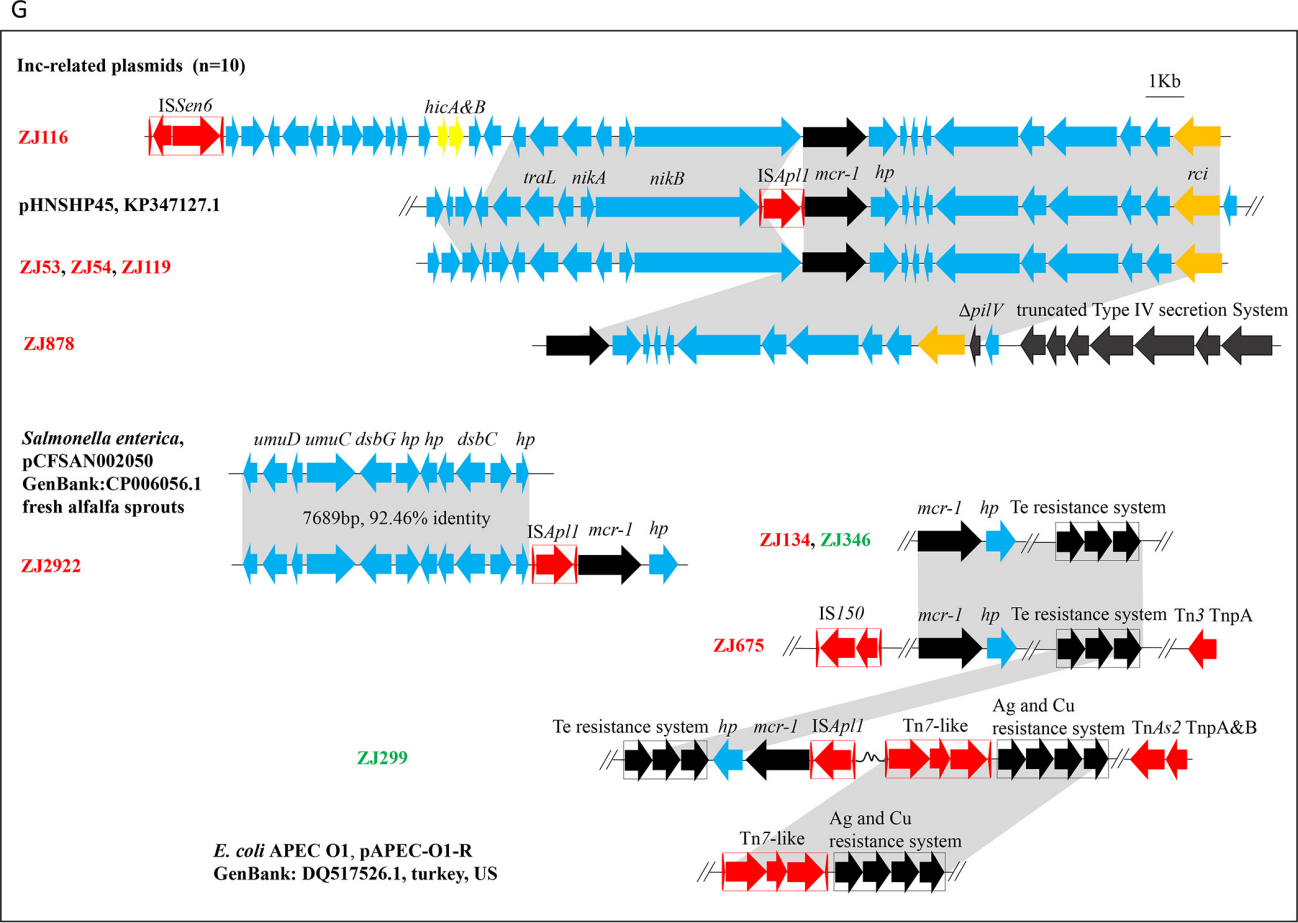
The diverse genetic environments of *mcr-1* in 80 E. coli isolates. In panels A to G, red fonts indicate isolates from inpatients with infections; green fonts denote isolates from feces of inpatients; and black fonts depict isolates from feces of healthy volunteers. APEC, avian pathogenic E. coli; ORF, open reading frame.

To characterize the mobile genetic elements and antibiotic resistance genes in the immediate proximity of *mcr-1*, we extracted the *mcr-1*-bearing contigs from 65 draft genomes and 15 complete genomes. Of the 65 contigs from draft genomes, 63 ranged from 12.1 kb to 141.2 kb in size; the remaining two were ~6.7 kb and ~8.3 kb in ZJ1682 and ZJ878, respectively (Table S1). We classified the genetic context into the following nine groups: an IncI2-related group (*n* = 29), an IncX4-related group (*n* = 28), an IncHI2-related group (*n* = 2), an IncP-related group (*n* = 2), an Incp0111-related group (*n* = 2), an IncY-related group (*n* = 1), an IncFIB-related group (*n* = 1), an non-Inc-related group (*n* = 10), and an chromosome-related group (*n* = 7). Overall, considerable diversity was found in the immediate environment of *mcr-1*. Compared to the previous reports, several novel genetic environments of *mcr-1* were found in this study, especially among those located on the chromosomes (Fig. 3).

### IncI2-related contigs.

The contigs from 29 isolates were IncI2-related (Fig. 2; see also Fig. S7 at https://doi.org/10.6084/m9.figshare.6281579.v1). The flanking sequences of *mcr-1* in all 29 isolates were very similar to sequences in plasmid pHNSHP45 (IncI2; KP347127.1), the first reported *mcr-1*-carrying plasmid in E. coli of swine origin in China. The IS*Apl1* insertion sequence is located immediately upstream of *mcr-1* in pHNSHP45. However, only three of our isolates (ZJ487, ZJ859, and ZJ3903) contained IS*Apl1* upstream of *mcr-1*, while the other 26 isolates showed no evidence of IS*Apl1* adjunct to *mcr-1* (Fig. 3A; see also Fig. S7 at https://doi.org/10.6084/m9.figshare.6281579.v1). Among the 26 isolates, 18 did not contain any insertion sequence and 8 contained IS elements other than IS*Apl1* (Fig. 3A). IS*Sen6*, initially found in Salmonella enterica (NC_006511), was present in *mcr-1*-carrying contigs in four isolates (ZJ1336, ZJ315, ZJ9, and ZJ257). IS*Kpn26*, initially found in K. pneumoniae (NC_016845), was detected in *mcr-1*-positive contigs in ZJ148, while IS*Ecp1* and IS*1294*, initially found in E. coli, were identified in *mcr-1*-positive contigs in ZJ33, ZJ89, and ZJ1635. Even though multiple ESBL genes were detected in isolate ZJ487, only *bla*_CTX-M-1_ was found to be located on the *mcr-1*-carrying plasmid shown by SMRT sequencing. *bla*_NDM-1_ and *bla*_SHV-12_ were found to be located on an ~54-kb plasmid, and *bla*_CTX-M-55_ and *bla*_OXA-1_ were found to be located on another, ~129-kb plasmid. In isolate ZJ33, *bla*_CTX-M-64_ flanked by IS*Ecp1* was shown to be located in the same *mcr-1*-carrying contig. Isolate ZJ859 harbored two IS*609* copies and one IS*2* copy in a *mcr-1*-carrying contig.

### IncX4-related contigs.

Twenty-eight contigs were associated with IncX4 (Fig. 2; see also Fig. S8 at https://doi.org/10.6084/m9.figshare.6281579.v1). The overall genetic contexts of these contigs showed similarity to plasmid pECGD-8-33 (IncX4; KX254343.1), and 26 of them did not show any evidence of insertion sequences (Fig. 3B; see also Fig. S8 at https://doi.org/10.6084/m9.figshare.6281579.v1). However, a truncated insertion sequence, IS*Kpn26*, was found upstream of *mcr-1* in ZJ1653, and IS*26* was found ~2 kb upstream of *mcr-1* in ZJ812. It is known that IS*26* is commonly associated with resistance genes in E. coli and other species (Fig. 3B) (41). However, no resistance genes were found on the *mcr-1*-carrying contigs of ZJ1653 or on the other IncX4-related contigs.

### IncHI2-related contigs.

Two *mcr-1*-positive contigs from isolates ZJ675 and ZJ650 were found to be IncHI2-related (Fig. 2; see also Fig. S9 at https://doi.org/10.6084/m9.figshare.6281579.v1), and the genetic context was found to be similar to that of pHNSHP45-2 (IncHI2; KU341381.1). The *mcr-1*-carrying contig in ZJ650 was 107,130 bp and did not harbor any IS elements or other antibiotic resistance genes. The *mcr-1*-positive contig in ZJ675 (141,207 bp) contains tellurium resistance system genes, i.e., a *ter* (tellurium resistance [Te^r^]) operon (*terZABCDEF*), conferring resistance to tellurite (42). In addition, IS*150* and Tn*3* were identified in the *mcr-1*-positive contig of ZJ675 (Fig. 3D; see also Fig. S9 at https://doi.org/10.6084/m9.figshare.6281579.v1).

### IncP-related contigs.

The genetic contexts of *mcr-1*-carrying contigs from isolates ZJ800 and ZJ623 are IncP-related and resemble DNA regions from plasmid pMCR_1511 (IncP; KX377410.1) (Fig. 2; see also Fig. S10 at https://doi.org/10.6084/m9.figshare.6281579.v1). IS*Apl1* is missing in the two *mcr-1*-carrying contigs (Fig. 3C); instead, IS*26* is distally present with *mcr-1*. No other antibiotic resistance genes were found in these two contigs.

### Incp0111-, IncY-, and IncFIB-related contigs.

The complete *mcr-1* plasmids in ZJ275 and ZJ276 possess the Incp0111 replicon (see Fig. S11 at https://doi.org/10.6084/m9.figshare.6281579.v1), while the complete *mcr-1*-positive plasmid in ZJ338 contains the IncY replicon (see Fig. S12 at https://doi.org/10.6084/m9.figshare.6281579.v1). The genetic contexts of Incp0111 and IncY are very similar, and *mcr-1* was flanked by IS*Apl1* at both ends, represented as IS*Apl1*–*mcr-1*–*hp*–IS*Apl1* (Fig. 3F; see also Fig. S11 at https://doi.org/10.6084/m9.figshare.6281579.v1), with the two IS*Apl1* elements in the same orientation. Inverse PCR using primers located within *mcr-1* was performed to detect the small circular form of this region in ZJ275 and ZJ276. PCR produced a 3,696-bp amplicon containing *mcr-1*, the region between *mcr-1* and two IS*Apl1* genes, and one intact IS*Apl1* element (data not shown), suggesting that recombination between the two IS*Apl1* copies may occur and may facilitate the transmission of *mcr-1*. One complete plasmid (81,972 bp) of ZJ859 possesses the IncFIB replicon (see Fig. S13 at https://doi.org/10.6084/m9.figshare.6281579.v1), in which *mcr-1* is flanked by IS*Apl1* and IS*26* upstream and IS*26* downstream (Fig. 3; see also Fig. S14 at https://doi.org/10.6084/m9.figshare.6281579.v1). Furthermore, genes corresponding to Tn*3*, IS*1A*, another two IS*26* elements, and a type IV secretion system were present in the plasmid (Fig. 3F). This plasmid also contains two additional antibiotic resistance genes, *bla*_TEM-1_ and *tetM*, which were associated with Tn*3* and proximal to *mcr-1*.

### Non-Inc-related contigs.

Ten *mcr-1*-positive contigs did not have replicon genes due to incomplete assembly (Fig. 2). As described previously, the possible Inc types of these contigs carrying plasmids were predicted through mapping the contigs to the reference plasmids (see Fig. S1 to S4 at https://doi.org/10.6084/m9.figshare.6281579.v1). The *mcr-1*-positive contigs of ZJ53, ZJ54, JZ116, ZJ119, and ZJ878 were similar to those of pHNSHP45 (IncI2) (see Fig. S1 at https://doi.org/10.6084/m9.figshare.6281579.v1); the *mcr-1*-positive contig of ZJ116 contains a copy of IS*Sen6*. In isolate ZJ2922, IS*Apl1*–*mcr-1*–*hp* was located downstream of a 7,689-bp segment that exhibited 92.5% nucleotide identity to the corresponding region of plasmid pCFSAN002050 from Salmonella enterica (GenBank accession number CP006056.1) (Fig. 3G). In isolates ZJ134, ZJ346, ZJ299, and ZJ675, the *mcr-1*-positive contigs contain the tellurium resistance system, which is commonly carried by IncHI2 or InHII plasmids (43). Moreover, a Tn*7*-like transposon element and two resistance systems for heavy metals, the *pco* (*COPR*) operon (*pcoEABCDRSE*) for copper resistance and the *sil* (*silR*) operon (*silESRCBAP*) for silver resistance, were identified upstream of IS*Apl1*–*mcr-1*–*hp* in isolate ZJ299, which is similar to the corresponding region of IncHI2 plasmid pAPEC-O1-R (DQ517526) from E. coli isolated from turkey originating from United States (44) (Fig. 3G).

### Chromosome-borne *mcr-1.*

*mcr-1* was located on the chromosome in seven isolates; six of the isolates were subjected to SMRT sequencing, and the remaining isolate, ZJ3951, was subjected to Illumina sequencing (Fig. 3E; see also Fig. S15 at https://doi.org/10.6084/m9.figshare.6281579.v1). The *mcr-1*-carrying contig of ZJ3951 is ~19.7 kb in size and similar to the chromosome of E. coli G27 from Belgium (accession number LOOY01000011.1) (Fig. 3E). In six isolates, ZJ40, ZJ167, ZJ250, ZJ393, ZJ1323, and ZJ1794, *mcr-1* was flanked by two IS*Apl1* elements, forming a unit of IS*Apl1*–*mcr-1*–*hp*–IS*Apl1* as observed in plasmids from ZJ275 and ZJ276. Interestingly, *mcr-1* was inserted in the same location in only two isolates, ZJ1323 and ZJ250, which belong to ST4014, and was located in the quite different regions in the other five isolates. All five isolates belong to different ST clades, and the integration sites of *mcr-1* on the chromosome were distinct (Fig. 3E), indicating that the mobile genetic unit of “IS*Apl1*–*mcr-1*–*hp*–IS*Apl1*” has been recently disseminated among E. coli isolates.

### *mcr-1* allelic variations.

Here, 10 MCR-1 variants (*mcr-1.2* to *mcr-1.11*) were deposited in NCBI, and in this study novel *mcr-1* variants were detected in six isolates (ZJ10, ZJ2431, ZJ299, ZJ3920, ZJ346, and ZJ1432) from various sources (Table S6). The allelic variants from isolates ZJ10, ZJ2431, and ZJ346 exhibited synonymous mutations at positions 18 (T→C), 27 (C→T), and 552 (C→T), respectively. Moreover, ZJ299 and ZJ3920 each harbored a single nucleotide mutation in *mcr-1*, resulting in nonsynonymous mutations at positions 145 (G→A, Gly→Ser), and 1423 (G→A, Val→Ile), respectively. Finally, isolate ZJ1432, which had two *mcr-1* copies in two different plasmids, contained a synonymous mutation at site 27 (C→T) in the *mcr-1* copy on the IncX4 plasmid. All the mutations were confirmed by Sanger sequencing (data not shown).

10.1128/mBio.00943-18.6 TABLE S6  Allelic variations of seven *mcr-1*-carrying isolates. Download TABLE S6, XLSX file, 0.03 MB. Copyright © 2018 Shen et al. 2018 Shen et al. This content is distributed under the terms of the Creative Commons Attribution 4.0 International license.

## DISCUSSION

In this study, 80 *mcr-1*-positive E. coli isolates of intestinal or extraintestinal sources from a single hospital in China were analyzed by a combination of Illumina and SMRT sequencing. We observed significant diversity not only in the genetic context of *mcr-1* but particularly in the host E. coli. The *mcr-1*-positive E. coli isolates are distributed throughout the four phylogroups (A, B1, B2, and D) (32) and represented by 50 ST clades, indicating a lack of clonal spread and implying no outbreak of *mcr-1*-positive E. coli in this hospital (Fig. 1). In a recent study conducted by another Chinese group, MCRPEC isolates of fecal sources were also found to be genetically diverse (45). However, a case of possible nosocomial transmission of *mcr-1*-positive E. coli was observed. The complete genomes of ZJ275 and ZJ276 revealed that the two isolates contain identical chromosomes and plasmids, with only 32 single nucleotide variants (SNVs) (4 single nucleotide polymorphisms [SNPs] and 28 indels) in the chromosome and 2 SNVs in the *mcr-1*-positive plasmid. It should be noted that the *mcr-1* gene was located on p0111 plasmids, which were specific to these two strains. Furthermore, ZJ275 and ZJ276 were isolated from the puncture fluid of a 76-year-old man and from the drainage fluid of a 38-year-old woman. The patients were kept in the same surgical intensive care unit during the same period and therefore, the possibility exists of nosocomial transmission of *mcr-1*-positive E. coli, highlighting the importance of hospital-acquired infections and hospital infection control programs (46).

Previous epidemiological studies indicated that groups A and B1 are usually composed of commensal strains or intestinal pathogenic strains (47) and groups B2 and D are associated with extraintestinal pathogenic strains (36, 48). However, in this study, no clear phylogenomic divisions were observed between intestinal strains and extraintestinal strains. Interestingly, examination of 14 extraintestinal isolates from bloodstream (*n* = 2) and urinary tract (*n* = 12) showed that only two isolates belonged to groups B2 and D (Fig. 1). Furthermore, analysis of virulence factors did not show any correlation between intestinal isolates and extraintestinal isolates (Fig. 1; see also Table S4 in the supplemental material). Although the data set is limited, this observation suggests that the intestinal strains may potentially cause systemic infections and serve as a source of nosocomial infections. In hospital settings, many patients are immunocompromised, have indwelling urinary catheters, and are exposed to numerous antimicrobials (Table S2), which might promote extraintestinal infection by antibiotic-resistant E. coli strains that are not normally considered extraintestinal pathogenic E. coli strains.

Acquisition of antibiotic resistance determinants through horizontal gene transfer is highly problematic. The presence of antibiotic resistance gene-linked transferable elements facilitates their spread among different clones and different bacterial species (49). *mcr-1* can be found in various plasmids and in different locations on the chromosomes of E. coli. The plasmids were classified into 8 Inc types by bioinformatics analysis, and the IncI2 (38.7%) and IncX4 (37.3%) plasmids were found to be the most prevalent types among the isolates, consistent with previous reports (21, 50, 51). IncI2 *mcr-1*-carrying plasmids were previously found to be dominant in E. coli-associated bloodstream infections (29). However, our study data indicate that neither IncI2 plasmids nor IncX4 plasmids were predominantly associated with intestinal or extraintestinal sources, patients (6/27 IncI2, 11/27 IncX4), or volunteers (8/17 IncI2, 5/17 IncX4). Interestingly, the genetic contexts of *mcr-1* were found to be highly divergent, and yet the majority of *mcr-1* genes were not adjacent to IS elements (17 were flanked with IS elements [Fig. 3]). IS*Apl1* was the most common IS element and was found to be adjacent to *mcr-1* at one end or both ends in 15 isolates. The presence of IS*Apl1* at the both ends of *mcr-1* makes the IS*Apl1*–*mcr-1*–*hp*–IS*Apl1* unit potentially active, as was evidenced by the circular form shown by inverse PCR, which can integrate into various locations on the chromosome and/or plasmids in a manner consistent with previous reports (52, 53). Furthermore, IS*Kpn26* was adjacent to *mcr-1* in isolates ZJ148 and ZJ1653, and several other IS elements such as IS*2*, IS*609*, IS*683*, IS*1294*, IS*150*, and IS*Sen6* were found in the *mcr-1*-positive contigs but not in locations immediately proximal to *mcr-1*. Whether these IS elements could contribute to the mobilization of *mcr-1* remains unknown, but their presence would enhance the transmission of *mcr-1* and facilitate its close association with other antibiotic resistance genes as seen in isolates ZJ134, ZJ33, ZJ487, ZJ26, and ZJ71 (41, 54, 55).

In this study, a strategy combining Illumina sequencing and SMRT sequencing was applied to decipher the genetic diversity of *mcr-1*-positive E. coli isolates. Although we successfully decoded the phylogenetic relationships, virulence factors, antibiotic resistance genes, and genetic contexts of *mcr-1* of 80 isolates, we could not definitively determine how many antibiotic resistance genes coexist with *mcr-1* on the same plasmids due to some incomplete assemblies (generated by short reads of Illumina sequencing). SMRT sequencing offers a definitive solution for deciphering individual bacterial isolates by virtue of its long reads, but the significantly higher cost limits its application for large numbers of samples. We therefore subjected 15 isolates to SMRT sequencing to complement the data set generated by Illumina sequencing. This combined-sequencing method revealed that *mcr-1* was located on the chromosome of 7 isolates and in the plasmids of 73 isolates. New sequencing technologies (such as nanopore sequencing) that generate long sequence reads may be used in future studies to decipher *mcr-1*-carrying MDR plasmids (56), which would facilitate our understanding of the cotransfer of *mcr-1* with other antimicrobial resistance genes in bacterial plasmids.

## MATERIALS AND METHODS

### Ethical approval.

Ethical approval of the study was granted by the Second Affiliated Hospital of Zhejiang University.

### Genome sequencing and assembly.

Genomic DNA of all *mcr-1*-positive isolates was extracted using a Wizard genomic DNA purification kit (Promega, Beijing, China), following the manufacturer’s instructions. Indexed Illumina sequencing libraries were prepared using a TruSeq DNA PCR-free sample preparation kit (Illumina Inc., San Diego, CA) following the standard protocol and were sequenced on an Illumina HiSeq 2500 platform according to the manufacturer’s protocols; the sequencing produced 250-bp paired-end reads (Bionova, Beijing, China). The draft genomes were assembled using the SPAdes algorithm (57) and CLC Genomics workbench 8.5 (CLC Bio, Aarhus, Denmark). To optimize the assembly of plasmids, Illumina reads of each isolates were reassembled by plasmidSPAdes, a program to assemble plasmids from whole-genome sequencing data (58). All the assemblies were further corrected by Pilon (59). The *mcr-1*-containing contigs for each isolate were extracted from the two assemblies, and the longer one was used for analysis of the genetic context of *mcr-1*.

Fifteen isolates (9 isolates that generated only short *mcr-1*-containing contigs in Illumina sequencing and 6 isolates that were able to cotransfer other antibiotic resistance genes with *mcr-1*) were further sequenced by the use of SMRT sequencing (60). Genomic DNA was sheared to 10 to 17 kb using Covaris g-tubes (Covaris) and converted into SMRTbell template libraries. The libraries were subsequently subjected to DNA size selection using a BluePippin instrument (Sage Science) to select the longest DNA fragments (lower size cutoff value of ~5 kb). Sequencing was performed on a PacBio RSII system using P6 polymerase binding and C4 sequencing kits with magnetic bead loading and 120-min acquisition (Sinobiocore, Beijing, China). Genome assemblies were performed using HGAP and Quiver as part of SMRTAnalysis version 2.3 by the use of the HGAP3 protocol and corrected using Pilon. Shotgun sequences of all 80 isolates have been deposited in the NCBI database (see below).

### Molecular epidemiology and analysis of virulence and antibiotic resistance genes.

Assembled genomes from Illumina and Pacbio sequencing were aligned, and a core-genome phylogenetic tree was generated by Parsnp in the Harvest package (61). MLST analysis and examinations of known virulence-associated genes and antibiotic resistance genes were carried out using pipeline SRST2, which takes Illumina reads as the input (62). Reference sequences of virulence genes and antibiotic resistance genes were from databases VFDB (63) and ARG-ANNOT (64), respectively. The tree and the molecular features of each isolate were visualized by the use of the online tool iTOL (65). Classification of strains to phylogenetic groups was performed by using assembled contigs according to a scheme described previously (32, 66).

### Analysis of *mcr-1* location.

The plasmid or chromosome location of the *mcr-1* gene in 80 E. coli isolates was first determined by S1-nuclease digestion, pulsed-field gel electrophoresis, and probing with the *mcr-1* DNA fragment as described previously (9). The location of *mcr-1* was further confirmed by sequence analysis. *mcr-1*-containing contigs generated by Illumina and Pacbio sequencing were examined for Inc types by PlasmidFinder (40). The *mcr-1*-carrying contigs encoding plasmid replicons were considered to come from plasmids, and all the contigs of the genome of each corresponding isolate were mapped to the reference plasmids by BLASTN (67). A closest reference plasmid was selected based on the mapping coverage to scaffold the *mcr-1*-carrying plasmid from each isolate.

### Analysis of the genetic context of *mcr-1.*

The *mcr-1*-carrying contigs were annotated by the use of the RAST annotation server (68). The insertion sequences were identified by ISfinder (69). For contigs in which *mcr-1* was immediately surrounded by IS elements or only the *mcr-1-*hp core region was observed, an inverse PCR assay using primers F-primer (TATTCTGTGCCGTGTATGTT) and R-primer (TATCAGGCTTGGTTGCTT) (annealing temperature, 55°C) located within the *mcr-1* gene was performed to determine the possible existence of a free circular form containing IS-flanked sequence.

### Additional material.

Supplemental figures can be found at figshare (https://doi.org/10.6084/m9.figshare.6281579.v1). These show the genetic contexts of *mcr-1* in the E. coli isolates from one hospital.

### Accession number(s).

Shotgun sequences of all 80 isolates have been deposited in the NCBI database (BioProject accession no. PRJNA331013 and BioSample accession no. SAMN05437795 to SAMN05437874), while 15 SMRT sequences have been deposited under other accession numbers (BioProject accession no. PRJNA380845 and BioSample accession no. SAMN06649969 to SAMN06649983).

## References

[1] Payne DJ, Gwynn MN, Holmes DJ, Pompliano DL 2007 Drugs for bad bugs: confronting the challenges of antibacterial discovery. Nat Rev Drug Discov 6:29–40. doi:10.1038/nrd2201. 17159923

[2] Alanis AJ 2005 Resistance to antibiotics: are we in the post-antibiotic era? Arch Med Res 36:697–705. doi:10.1016/j.arcmed.2005.06.009. 16216651

[3] Fears R, ter Meulen V 2014 What do we need to do to tackle antimicrobial resistance? Lancet Glob Health 2:e11–e12. doi:10.1016/S2214-109X(13)70086-X. 25104623

[4] Sprenger M, Fukuda K 2016 Antimicrobial resistance. New mechanisms, new worries. Science 351:1263–1264. doi:10.1126/science.aad9450. 26989235

[5] Munoz-Price LS, Poirel L, Bonomo RA, Schwaber MJ, Daikos GL, Cormican M, Cornaglia G, Garau J, Gniadkowski M, Hayden MK, Kumarasamy K, Livermore DM, Maya JJ, Nordmann P, Patel JB, Paterson DL, Pitout J, Villegas MV, Wang H, Woodford N, Quinn JP 2013 Clinical epidemiology of the global expansion of *Klebsiella pneumoniae* carbapenemases. Lancet Infect Dis 13:785–796. doi:10.1016/S1473-3099(13)70190-7. 23969216 PMC4673667

[6] Kumarasamy KK, Toleman MA, Walsh TR, Bagaria J, Butt F, Balakrishnan R, Chaudhary U, Doumith M, Giske CG, Irfan S, Krishnan P, Kumar AV, Maharjan S, Mushtaq S, Noorie T, Paterson DL, Pearson A, Perry C, Pike R, Rao B, Ray U, Sarma JB, Sharma M, Sheridan E, Thirunarayan MA, Turton J, Upadhyay S, Warner M, Welfare W, Livermore DM, Woodford N 2010 Emergence of a new antibiotic resistance mechanism in India, Pakistan, and the UK: a molecular, biological, and epidemiological study. Lancet Infect Dis 10:597–602. doi:10.1016/S1473-3099(10)70143-2. 20705517 PMC2933358

[7] Evans BA, Amyes SG 2014 OXA beta-lactamases. Clin Microbiol Rev 27:241–263. doi:10.1128/CMR.00117-13. 24696435 PMC3993105

[8] Bialvaei AZ, Samadi Kafil H 2015 Colistin, mechanisms and prevalence of resistance. Curr Med Res Opin 31:707–721. doi:10.1185/03007995.2015.1018989. 25697677

[9] Liu YY, Wang Y, Walsh TR, Yi LX, Zhang R, Spencer J, Doi Y, Tian G, Dong B, Huang X, Yu LF, Gu D, Ren H, Chen X, Lv L, He D, Zhou H, Liang Z, Liu JH, Shen J 2016 Emergence of plasmid-mediated colistin resistance mechanism MCR-1 in animals and human beings in China: a microbiological and molecular biological study. Lancet Infect Dis 16:161–168. 26603172 10.1016/S1473-3099(15)00424-7

[10] Falgenhauer L, Waezsada SE, Yao Y, Imirzalioglu C, Käsbohrer A, Roesler U, Michael GB, Schwarz S, Werner G, Kreienbrock L, Chakraborty T; RESET Consortium 2016 Colistin resistance gene mcr-1 in extended-spectrum β-lactamase-producing and carbapenemase-producing Gram-negative bacteria in Germany. Lancet Infect Dis 16:282–283. doi:10.1016/S1473-3099(16)00009-8. 26774242

[11] Li A, Yang Y, Miao M, Chavda KD, Mediavilla JR, Xie X, Feng P, Tang YW, Kreiswirth BN, Chen L, Du H 2016 Complete sequences of *mcr-1*-harboring plasmids from extended-spectrum-beta-lactamase- and carbapenemase-producing Enterobacteriaceae. Antimicrob Agents Chemother 60:4351–4354. doi:10.1128/AAC.00550-16. 27090180 PMC4914624

[12] Yao X, Doi Y, Zeng L, Lv L, Liu JH 2016 Carbapenem-resistant and colistin-resistant *Escherichia coli* co-producing NDM-9 and MCR-1. Lancet Infect Dis 16:288–289. doi:10.1016/S1473-3099(16)00057-8. 26842777

[13] Wang Y, Zhang R, Li J, Wu Z, Yin W, Schwarz S, Tyrrell JM, Zheng Y, Wang S, Shen Z, Liu Z, Liu J, Lei L, Li M, Zhang Q, Wu C, Zhang Q, Wu Y, Walsh TR, Shen J 2017 Comprehensive resistome analysis reveals the prevalence of NDM and MCR-1 in Chinese poultry production. Nat Microbiol 2:16260. doi:10.1038/nmicrobiol.2016.260. 28165472

[14] Ochman H, Lawrence JG, Groisman EA 2000 Lateral gene transfer and the nature of bacterial innovation. Nature 405:299–304. doi:10.1038/35012500. 10830951

[15] Toleman MA, Bennett PM, Walsh TR 2006 ISCR elements: novel gene-capturing systems of the 21st century? Microbiol Mol Biol Rev 70:296–316. doi:10.1128/MMBR.00048-05. 16760305 PMC1489542

[16] Poirel L, Dortet L, Bernabeu S, Nordmann P 2011 Genetic features of *bla*_NDM-1_-positive Enterobacteriaceae. Antimicrob Agents Chemother 55:5403–5407. doi:10.1128/AAC.00585-11. 21859933 PMC3195013

[17] McGann P, Snesrud E, Maybank R, Corey B, Ong AC, Clifford R, Hinkle M, Whitman T, Lesho E, Schaecher KE 2016 *Escherichia coli* harboring *mcr-1* and *bla*_CTX-M_ on a novel IncF plasmid: first report of *mcr-1* in the United States. Antimicrob Agents Chemother 60:4420–4421. doi:10.1128/AAC.01103-16. 27230792 PMC4914657

[18] Pham Thanh D, Thanh Tuyen H, Nguyen Thi Nguyen T, Chung The H, Wick RR, Thwaites GE, Baker S, Holt KE 2016 Inducible colistin resistance via a disrupted plasmid-borne *mcr-1* gene in a 2008 Vietnamese Shigella sonnei isolate. J Antimicrob Chemother 71:2314–2317. doi:10.1093/jac/dkw173. 27246235 PMC4954929

[19] Poirel L, Kieffer N, Brink A, Coetze J, Jayol A, Nordmann P 2016 Genetic features of MCR-1-producing colistin-resistant *Escherichia coli* isolates in South Africa. Antimicrob Agents Chemother 60:4394–4397. doi:10.1128/AAC.00444-16. 27161623 PMC4914673

[20] Xavier BB, Lammens C, Butaye P, Goossens H, Malhotra-Kumar S 2016 Complete sequence of an IncFII plasmid harbouring the colistin resistance gene *mcr-1* isolated from Belgian pig farms. J Antimicrob Chemother 71:2342–2344. doi:10.1093/jac/dkw191. 27261261

[21] Sun J, Li XP, Yang RS, Fang LX, Huo W, Li SM, Jiang P, Liao XP, Liu YH 2016 Complete nucleotide sequence of an IncI2 plasmid coharboring *bla*_CTX-M-55_ and *mcr-1*. Antimicrob Agents Chemother 60:5014–5017. doi:10.1128/AAC.00774-16. 27216063 PMC4958226

[22] Gao R, Wang Q, Li P, Li Z, Feng Y 2016 Genome sequence and characteristics of plasmid pWH12, a variant of the *mcr-1*-harbouring plasmid pHNSHP45, from the multi-drug resistant *E. coli*. Virulence 7:732–735. doi:10.1080/21505594.2016.1193279. 27221541 PMC4991314

[23] Zhi C, Lv L, Yu LF, Doi Y, Liu JH 2016 Dissemination of the mcr-1 colistin resistance gene. Lancet Infect Dis 16:292–293. 26973307 10.1016/S1473-3099(16)00063-3

[24] Li R, Xie M, Zhang J, Yang Z, Liu L, Liu X, Zheng Z, Chan EW, Chen S 2017 Genetic characterization of *mcr-1*-bearing plasmids to depict molecular mechanisms underlying dissemination of the colistin resistance determinant. J Antimicrob Chemother 72:393–401. doi:10.1093/jac/dkw411. 28073961

[25] Yu CY, Ang GY, Chong TM, Chin PS, Ngeow YF, Yin WF, Chan KG 2017 Complete genome sequencing revealed novel genetic contexts of the *mcr-1* gene in *Escherichia coli* strains. J Antimicrob Chemother 72:1253–1255. doi:10.1093/jac/dkw541. 28031273

[26] Li R, Xie M, Lv J, Wai-Chi Chan E, Chen S 2017 Complete genetic analysis of plasmids carrying *mcr-1* and other resistance genes in an *Escherichia coli* isolate of animal origin. J Antimicrob Chemother 72:696–699. doi:10.1093/jac/dkw509. 27999050

[27] Sun J, Fang LX, Wu Z, Deng H, Yang RS, Li XP, Li SM, Liao XP, Feng Y, Liu YH 2017 Genetic analysis of the IncX4 plasmids: implications for a unique pattern in the *mcr-1* acquisition. Sci Rep 7:424. doi:10.1038/s41598-017-00095-x. 28336940 PMC5428312

[28] Hasman H, Hammerum AM, Hansen F, Hendriksen RS, Olesen B, Agersø Y, Zankari E, Leekitcharoenphon P, Stegger M, Kaas RS, Cavaco LM, Hansen DS, Aarestrup FM, Skov RL 2015 Detection of *mcr-1* encoding plasmid-mediated colistin-resistant *Escherichia coli* isolates from human bloodstream infection and imported chicken meat, Denmark 2015. Euro Surveill 20. doi:10.2807/1560-7917.ES.2015.20.49.30085. 26676364

[29] Quan J, Li X, Chen Y, Jiang Y, Zhou Z, Zhang H, Sun L, Ruan Z, Feng Y, Akova M, Yu Y 2017 Prevalence of *mcr-1* in *Escherichia coli* and *Klebsiella pneumoniae* recovered from bloodstream infections in China: a multicentre longitudinal study. Lancet Infect Dis 17:400–410. doi:10.1016/S1473-3099(16)30528-X. 28139430

[30] Wang Y, Tian GB, Zhang R, Shen Y, Tyrrell JM, Huang X, Zhou H, Lei L, Li HY, Doi Y, Fang Y, Ren H, Zhong LL, Shen Z, Zeng KJ, Wang S, Liu JH, Wu C, Walsh TR, Shen J 2017 Prevalence, risk factors, outcomes, and molecular epidemiology of mcr-1-positive Enterobacteriaceae in patients and healthy adults from China: an epidemiological and clinical study. Lancet Infect Dis 17:390–399. doi:10.1016/S1473-3099(16)30527-8. 28139431

[31] Escobar-Páramo P, Le Menac’h A, Le Gall T, Amorin C, Gouriou S, Picard B, Skurnik D, Denamur E 2006 Identification of forces shaping the commensal *Escherichia coli* genetic structure by comparing animal and human isolates. Environ Microbiol 8:1975–1984. doi:10.1111/j.1462-2920.2006.01077.x. 17014496

[32] Clermont O, Bonacorsi S, Bingen E 2000 Rapid and simple determination of the *Escherichia coli* phylogenetic group. Appl Environ Microbiol 66:4555–4558. doi:10.1128/AEM.66.10.4555-4558.2000. 11010916 PMC92342

[33] Tartof SY, Solberg OD, Manges AR, Riley LW 2005 Analysis of a uropathogenic *Escherichia coli* clonal group by multilocus sequence typing. J Clin Microbiol 43:5860–5864. doi:10.1128/JCM.43.12.5860-5864.2005. 16333067 PMC1317175

[34] Oteo J, Diestra K, Juan C, Bautista V, Novais A, Pérez-Vázquez M, Moyá B, Miró E, Coque TM, Oliver A, Cantón R, Navarro F, Campos J; Spanish Network in Infectious Pathology Project (REIPI) 2009 Extended-spectrum beta-lactamase-producing *Escherichia coli* in Spain belong to a large variety of multilocus sequence typing types, including ST10 complex/A, ST23 complex/A and ST131/B2. Int J Antimicrob Agents 34:173–176. doi:10.1016/j.ijantimicag.2009.03.006. 19464856

[35] Cortés P, Blanc V, Mora A, Dahbi G, Blanco JE, Blanco M, López C, Andreu A, Navarro F, Alonso MP, Bou G, Blanco J, Llagostera M 2010 Isolation and characterization of potentially pathogenic antimicrobial-resistant *Escherichia coli* strains from chicken and pig farms in Spain. Appl Environ Microbiol 76:2799–2805. doi:10.1128/AEM.02421-09. 20228098 PMC2863447

[36] Salipante SJ, Roach DJ, Kitzman JO, Snyder MW, Stackhouse B, Butler-Wu SM, Lee C, Cookson BT, Shendure J 2015 Large-scale genomic sequencing of extraintestinal pathogenic *Escherichia coli* strains. Genome Res 25:119–128. doi:10.1101/gr.180190.114. 25373147 PMC4317167

[37] Adams-Sapper S, Diep BA, Perdreau-Remington F, Riley LW 2013 Clonal composition and community clustering of drug-susceptible and -resistant *Escherichia coli* isolates from bloodstream infections. Antimicrob Agents Chemother 57:490–497. doi:10.1128/AAC.01025-12. 23147723 PMC3535975

[38] Gibreel TM, Dodgson AR, Cheesbrough J, Fox AJ, Bolton FJ, Upton M 2012 Population structure, virulence potential and antibiotic susceptibility of uropathogenic *Escherichia coli* from northwest England. J Antimicrob Chemother 67:346–356. doi:10.1093/jac/dkr451. 22028202

[39] Petty NK, Ben Zakour NL, Stanton-Cook M, Skippington E, Totsika M, Forde BM, Phan MD, Gomes Moriel D, Peters KM, Davies M, Rogers BA, Dougan G, Rodriguez-Baño J, Pascual A, Pitout JD, Upton M, Paterson DL, Walsh TR, Schembri MA, Beatson SA 2014 Global dissemination of a multidrug resistant *Escherichia coli* clone. Proc Natl Acad Sci U S A 111:5694–5699. doi:10.1073/pnas.1322678111. 24706808 PMC3992628

[40] Carattoli A, Zankari E, García-Fernández A, Voldby Larsen M, Lund O, Villa L, Møller Aarestrup F, Hasman H 2014 In silico detection and typing of plasmids using PlasmidFinder and plasmid multilocus sequence typing. Antimicrob Agents Chemother 58:3895–3903. doi:10.1128/AAC.02412-14. 24777092 PMC4068535

[41] Harmer CJ, Hall RM 2016 IS*26*-mediated formation of transposons carrying antibiotic resistance genes. mSphere 1:e00038-16. doi:10.1128/mSphere.00038-16. PMC4894685 27303727

[42] Whelan KF, Sherburne RK, Taylor DE 1997 Characterization of a region of the IncHI2 plasmid R478 which protects *Escherichia coli* from toxic effects specified by components of the tellurite, phage, and colicin resistance cluster. J Bacteriol 179:63–71. doi:10.1128/jb.179.1.63-71.1997. 8981981 PMC178662

[43] Taylor DE 1999 Bacterial tellurite resistance. Trends Microbiol 7:111–115. doi:10.1016/S0966-842X(99)01454-7. 10203839

[44] Johnson TJ, Wannemeuhler YM, Scaccianoce JA, Johnson SJ, Nolan LK 2006 Complete DNA sequence, comparative genomics, and prevalence of an IncHI2 plasmid occurring among extraintestinal pathogenic *Escherichia coli* isolates. Antimicrob Agents Chemother 50:3929–3933. doi:10.1128/AAC.00569-06. 16940062 PMC1635206

[45] Zhong LL, Phan HTT, Shen C, Doris-Vihta K, Sheppard AE, Huang X, Zeng KJ, Li HY, Zhang XF, Patil S, Crook DW, Walker AS, Xing Y, Lin JL, Feng LQ, Doi Y, Xia Y, Stoesser N, Tian GB 2018 High rates of human fecal carriage of mcr-1-positive multi-drug resistant Enterobacteriaceae isolates emerge in China in association with successful plasmid families. Clin Infect Dis 66:676–685. doi:10.1093/cid/cix885. 29040419 PMC5848316

[46] Peleg AY, Hooper DC 2010 Hospital-acquired infections due to gram-negative bacteria. N Engl J Med 362:1804–1813. doi:10.1056/NEJMra0904124. 20463340 PMC3107499

[47] von Mentzer A, Connor TR, Wieler LH, Semmler T, Iguchi A, Thomson NR, Rasko DA, Joffre E, Corander J, Pickard D, Wiklund G, Svennerholm AM, Sjöling Å, Dougan G 2014 Identification of enterotoxigenic Escherichia coli (ETEC) clades with long-term global distribution. Nat Genet 46:1321–1326. doi:10.1038/ng.3145. 25383970

[48] Picard B, Garcia JS, Gouriou S, Duriez P, Brahimi N, Bingen E, Elion J, Denamur E 1999 The link between phylogeny and virulence in *Escherichia coli* extraintestinal infection. Infect Immun 67:546–553. 9916057 10.1128/iai.67.2.546-553.1999 PMC96353

[49] Martínez JL, Coque TM, Lanza VF, de la Cruz F, Baquero F 2017 Genomic and metagenomic technologies to explore the antibiotic resistance mobilome. Ann N Y Acad Sci 1388:26–41. doi:10.1111/nyas.13282. 27861983

[50] Fernandes MR, McCulloch JA, Vianello MA, Moura Q, Pérez-Chaparro PJ, Esposito F, Sartori L, Dropa M, Matté MH, Lira DP, Mamizuka EM, Lincopan N 2016 First report of the globally disseminated IncX4 plasmid carrying the *mcr-1* gene in a colistin-resistant *Escherichia coli* sequence type 101 isolate from a human infection in Brazil. Antimicrob Agents Chemother 60:6415–6417. doi:10.1128/AAC.01325-16. 27503650 PMC5038249

[51] Falgenhauer L, Waezsada SE, Gwozdzinski K, Ghosh H, Doijad S, Bunk B, Spröer C, Imirzalioglu C, Seifert H, Irrgang A, Fischer J, Guerra B, Käsbohrer A, Overmann J, Goesmann A, Chakraborty T 2016 Chromosomal locations of *mcr-1* and *bla*_CTX-M-15_ in fluoroquinolone-resistant *Escherichia coli* ST410. Emerg Infect Dis 22:1689–1691. doi:10.3201/eid2209.160692. 27322919 PMC4994348

[52] Snesrud E, Ong AC, Corey B, Kwak YI, Clifford R, Gleeson T, Wood S, Whitman TJ, Lesho EP, Hinkle M, McGann P 2017 Analysis of serial isolates of *mcr-1*-positive *Escherichia coli* reveals a highly active IS*Apl1* transposon. Antimicrob Agents Chemother 61:e00056-17. doi:10.1128/AAC.00056-17. 28223389 PMC5404521

[53] Snesrud E, He S, Chandler M, Dekker JP, Hickman AB, McGann P, Dyda F 2016 A model for transposition of the colistin resistance gene *mcr-1* by ISApl1. Antimicrob Agents Chemother 60:6973–6976. doi:10.1128/AAC.01457-16. 27620479 PMC5075121

[54] Haas M, Rak B 2002 *Escherichia coli* insertion sequence IS150: transposition via circular and linear intermediates. J Bacteriol 184:5833–5841. doi:10.1128/JB.184.21.5833-5841.2002. 12374815 PMC135391

[55] Gonçalves GA, Oliveira PH, Gomes AG, Prather KL, Lewis LA, Prazeres DM, Monteiro GA 2014 Evidence that the insertion events of IS*2* transposition are biased towards abrupt compositional shifts in target DNA and modulated by a diverse set of culture parameters. Appl Microbiol Biotechnol 98:6609–6619. doi:10.1007/s00253-014-5695-6. 24769900

[56] Li R, Xie M, Dong N, Lin D, Yang X, Wong MHY, Chan EW, Chen S 2018 Efficient generation of complete sequences of MDR-encoding plasmids by rapid assembly of MinION barcoding sequencing data. Gigascience 7:1–9. doi:10.1093/gigascience/gix132. PMC5848804 29325009

[57] Bankevich A, Nurk S, Antipov D, Gurevich AA, Dvorkin M, Kulikov AS, Lesin VM, Nikolenko SI, Pham S, Prjibelski AD, Pyshkin AV, Sirotkin AV, Vyahhi N, Tesler G, Alekseyev MA, Pevzner PA 2012 SPAdes: a new genome assembly algorithm and its applications to single-cell sequencing. J Comput Biol 19:455–477. doi:10.1089/cmb.2012.0021. 22506599 PMC3342519

[58] Antipov D, Hartwick N, Shen M, Raiko M, Lapidus A, Pevzner PA 2016 plasmidSPAdes: assembling plasmids from whole genome sequencing data. Bioinformatics 32:3380–3387. doi:10.1093/bioinformatics/btw493. 27466620

[59] Walker BJ, Abeel T, Shea T, Priest M, Abouelliel A, Sakthikumar S, Cuomo CA, Zeng Q, Wortman J, Young SK, Earl AM 2014 Pilon: an integrated tool for comprehensive microbial variant detection and genome assembly improvement. PLoS One 9:e112963. doi:10.1371/journal.pone.0112963. 25409509 PMC4237348

[60] Eid J, Fehr A, Gray J, Luong K, Lyle J, Otto G, Peluso P, Rank D, Baybayan P, Bettman B, Bibillo A, Bjornson K, Chaudhuri B, Christians F, Cicero R, Clark S, Dalal R, Dewinter A, Dixon J, Foquet M, Gaertner A, Hardenbol P, Heiner C, Hester K, Holden D, Kearns G, Kong X, Kuse R, Lacroix Y, Lin S, Lundquist P, Ma C, Marks P, Maxham M, Murphy D, Park I, Pham T, Phillips M, Roy J, Sebra R, Shen G, Sorenson J, Tomaney A, Travers K, Trulson M, Vieceli J, Wegener J, Wu D, Yang A, Zaccarin D, et al. 2009 Real-time DNA sequencing from single polymerase molecules. Science 323:133–138. doi:10.1126/science.1162986. 19023044

[61] Treangen TJ, Ondov BD, Koren S, Phillippy AM 2014 The Harvest suite for rapid core-genome alignment and visualization of thousands of intraspecific microbial genomes. Genome Biol 15:524. doi:10.1186/PREACCEPT-2573980311437212. 25410596 PMC4262987

[62] Inouye M, Dashnow H, Raven LA, Schultz MB, Pope BJ, Tomita T, Zobel J, Holt KE 2014 SRST2: rapid genomic surveillance for public health and hospital microbiology labs. Genome Med 6:90. doi:10.1186/s13073-014-0090-6. 25422674 PMC4237778

[63] Chen L, Zheng D, Liu B, Yang J, Jin Q 2016 VFDB 2016: hierarchical and refined dataset for big data analysis—10 years on. Nucleic Acids Res 44:D694–D697. doi:10.1093/nar/gkv1239. 26578559 PMC4702877

[64] Gupta SK, Padmanabhan BR, Diene SM, Lopez-Rojas R, Kempf M, Landraud L, Rolain JM 2014 ARG-ANNOT, a new bioinformatic tool to discover antibiotic resistance genes in bacterial genomes. Antimicrob Agents Chemother 58:212–220. doi:10.1128/AAC.01310-13. 24145532 PMC3910750

[65] Letunic I, Bork P 2016 Interactive tree of life (iTOL) v3: an online tool for the display and annotation of phylogenetic and other trees. Nucleic Acids Res 44:W242–W245. doi:10.1093/nar/gkw290. 27095192 PMC4987883

[66] Wirth T, Falush D, Lan R, Colles F, Mensa P, Wieler LH, Karch H, Reeves PR, Maiden MC, Ochman H, Achtman M 2006 Sex and virulence in *Escherichia coli*: an evolutionary perspective. Mol Microbiol 60:1136–1151. doi:10.1111/j.1365-2958.2006.05172.x. 16689791 PMC1557465

[67] Camacho C, Coulouris G, Avagyan V, Ma N, Papadopoulos J, Bealer K, Madden TL 2009 Blast+: architecture and applications. BMC Bioinformatics 10:421. doi:10.1186/1471-2105-10-421. 20003500 PMC2803857

[68] Brettin T, Davis JJ, Disz T, Edwards RA, Gerdes S, Olsen GJ, Olson R, Overbeek R, Parrello B, Pusch GD, Shukla M, Thomason JA III, Stevens R, Vonstein V, Wattam AR, Xia F 2015 RASTtk: a modular and extensible implementation of the RAST algorithm for building custom annotation pipelines and annotating batches of genomes. Sci Rep 5:8365. doi:10.1038/srep08365. 25666585 PMC4322359

[69] Siguier P, Perochon J, Lestrade L, Mahillon J, Chandler M 2006 ISfinder: the reference centre for bacterial insertion sequences. Nucleic Acids Res 34:D32–D36. doi:10.1093/nar/gkj014. 16381877 PMC1347377

